# Effects of different physical activity interventions on children with attention-deficit/hyperactivity disorder: A network meta-analysis of randomized controlled trials

**DOI:** 10.3389/fnins.2023.1139263

**Published:** 2023-03-20

**Authors:** Dong Li, Deng Wang, Wenlai Cui, Jin Yan, Wanli Zang, Chenmu Li

**Affiliations:** ^1^School of Physical Education and Sports Science, Guangzhou Sport University, Guangzhou, China; ^2^LFE Research Group, Department of Health and Human Performance, Universidad Politécnica de Madrid, Madrid, Spain; ^3^School of Dance and Martial Arts, Capital University of Physical Education and Sports, Beijing, China; ^4^Centre for Active Living and Learning, University of Newcastle, Callaghan, NSW, Australia; ^5^Postgraduate School, University of Harbin Sport, Harbin, China

**Keywords:** physical activity, children, ADHD, neurodevelopmental disorders, network meta-analysis

## Abstract

**Background:**

Previous studies have shown that physical activity interventions positively affect core symptoms and executive functioning in children with attention-deficit/hyperactivity disorder (ADHD). However, comparisons between different physical activity interventions still need to be made. This study is the first to analyze the effects of 10 different types of physical activity on children with ADHD through a network meta-analysis.

**Methods:**

PubMed, Embase, Web of Science, and Cochrane Library databases were searched for randomized controlled trials on the effects of physical activity interventions on children with ADHD. The search time frame was from database creation to October 2022. Two investigators independently performed literature screening, extraction, and quality assessment. Network meta-analysis was performed with Stata 15.1.

**Results:**

A total of 31 studies were included, and the results indicated that perceptual-motor training was the most effective in terms of motor ability and working memory (SUCRA = 82.7 and 73.3%, respectively). For attention problems and cognitive flexibility, aquatic exercise was the most effective (SUCRA = 80.9 and 86.6%, respectively). For social problems, horsemanship was the most effective (SUCRA = 79.4%). For inhibition switching, cognitive-motor training was the most effective (SUCRA = 83.5%).

**Conclusion:**

Our study revealed that aquatic exercise and perceptual-motor training had a superior overall performance. However, the effects of various physical activity interventions on different indicators in children with ADHD can vary depending on the individual and the intervention’s validity. To ensure an appropriate physical activity intervention is selected, it is important to assess the severity of symptoms exhibited by children with ADHD beforehand.

## 1. Introduction

Attention-deficit/hyperactivity disorder (ADHD) is a neurodevelopmental disorder that affects approximately 7.2% of children worldwide (Thomas et al., 2015; Thapar et al., 2017). Its primary characteristics are inattention, impulsivity, and hyperactivity (American Psychiatric Association [APA], 2013), and can be divided into three distinct subtypes: inattentive, hyperactive-impulsive, and combined inattentive and hyperactive-impulsive (American Psychiatric Association [APA], 2013). It has been shown that children with ADHD often develop diverse problems, including sleep disturbances, distractibility, motor deficits, decreased social skills, and decreased academic performance (Kim et al., 2011; Konicarova et al., 2014; Schneider et al., 2016; Yu et al., 2019). These issues are also persistent, frequently remaining when patients reach puberty and adulthood (Edition, 2013). Therefore, it is highly detrimental to the development of pediatric patients and hurts their physical and mental health, academic growth, and socialization process.

Given this, the treatment of pediatric patients with ADHD is of utmost importance. The most commonly used treatment modality is medication, such as methylphenidate (MPH) (Barkley and Poillion, 1994; Welsch et al., 2021), but it may cause side effects such as headache, stomach pain, and decreased appetite (De Sousa and Kalra, 2012). Meanwhile, in the past two decades, non-pharmacological interventions for ADHD have been rapidly developed and used (Cortese et al., 2022), such as physical activity interventions, neurofeedback interventions, and cognitive interventions (Jensen and Kenny, 2004; Sánchez-López et al., 2015; Sani et al., 2022), due to concerns about the side effects and long-term effects of pharmacological treatments (Coghill, 2019). Physical activity interventions, in particular, have gained traction due to their lower cost, ease of implementation, capacity to improve physical fitness, and additional benefits (Cornelius et al., 2017).

Previous research has uncovered a strong link between physical activity and various functions in individuals with ADHD. Barnard-Brak et al. (2011) utilized data from the Early Childhood Longitudinal Study, Kindergarten cohort (ECLS-K) to demonstrate that structured physical activity was associated with a decrease in ADHD symptoms over time. This may be due to the stimulation of the catecholamine system, which is known to be impaired in individuals with ADHD (Barnard-Brak et al., 2011).

A recent study conducted by Fard et al. (2022) investigated the impact of physical activity on the physical and mental health of children and adolescents with ADHD, with self-esteem as a moderating factor. The results indicated that physical activity and health levels are integral components of well-being for this population and that self-esteem could be a potential mediator for the connection between physical activity and health outcomes (Fard et al., 2022). Some previous meta-analyzes have also shown evidence of better efficacy of physical activity in patients with ADHD. Cerrillo-Urbina et al. (2015) explored the impact of physical activity on core symptoms of attention, impulsivity, anxiety, and executive functioning in patients with ADHD. The results showed that physical activity was more effective than non-physical activity, particularly aerobic exercise (Cerrillo-Urbina et al., 2015). Zang (2019) assessed the effects of physical activity interventions compared to non-physical activity interventions in children with ADHD. The findings indicated that physical activity interventions had a significant positive effect on anxiety and depression, aggressive behavior, thinking, and social problems in children with ADHD (Zang, 2019). Lambez et al. (2020) performed a meta-analysis evaluating the effects of non-pharmacological treatments for ADHD on cognitive functioning. The interventions studied included neurofeedback, cognitive behavioral therapy, cognitive training, and physical exercise. Physical exercise was found to have the greatest mean effect size, particularly for inhibition (Lambez et al., 2020). Seiffer et al. (2022) studied the efficacy of moderate to vigorous exercise (MVPA) on children with attention deficit hyperactivity disorder (ADHD), focusing on the intensity component of physical activity. The study indicated that MVPA was the most effective treatment for ADHD and that it might be used as an alternative (Seiffer et al., 2022). Collectively, these findings suggest that physical exercise may be an effective treatment option for ADHD patients.

However, previous meta-analyzes have largely compared physical and non-physical activity, without examined the potential distinctions between different types of physical activity interventions. The types of physical activity are diverse and include many types of aquatic exercise, ball games, mind-body exercise, and high-intensity interval training. Therefore, what specific types of physical activity provide the most significant benefit to pediatric patients with ADHD? Through a network meta-analysis of randomized controlled trial studies of physical activity in pediatric patients with ADHD, this study provides valuable information for selecting the best physical activity for treating pediatric patients with ADHD.

## 2. Materials and methods

### 2.1. Protocol and registration

The meta-analysis was conducted using the Cochrane Handbook for Systematic Reviews of Interventions, and the findings were reported according to the Preferred Reporting Items for Systematic Reviews and Meta-Analyzes (PRISMA) statement (Higgins et al., 2019; Page et al., 2021). This network meta-analysis was prospectively registered in PROSPERO (CRD 42022363255).

### 2.2. Data sources and search strategy

We conducted a comprehensive search of four databases (PubMed, Web of Science, Embase, and the Cochrane Library) to identify relevant studies. Search strings included physical activity interventions, age ranges, and outcomes related to patients with ADHD. The search was performed up to October 2022. Supplementary Appendix A shows the detailed search strings for this search.

### 2.3. Study selection

Following guidelines, two authors (DW and DL) independently assessed the search results and vetted the publications retrieved from databases and reference lists. The titles and abstracts of the research were first used to determine their relevance. Then, relevant full-text studies were retrieved and evaluated for inclusion. Any disagreements were resolved through discussion and consensus.

### 2.4. Inclusion and exclusion criteria

This systematic review employed specified inclusion criteria. Each study met the following criteria:

(1) Only randomized controlled trials were included, and observational and cross-sectional studies were excluded.
(2) The range of age participants in the sample must be 18 years or less.
(3) The physical activity intervention had to contain a sports or physical activity component. Studies without physical activity intervention were excluded.
(4) The study must report data on indicators of motor skills, attention problems, social problems, cognitive flexibility, inhibitory switching, and working memory in children with ADHD before and after the intervention. Studies that do not report on these indicators must be excluded.
(5) Presented original data.
(6) We only analyzed papers written in English and excluded papers written in other languages.

### 2.5. Data extraction

The data were extracted to a standardized Excel spreadsheet. Two authors collected the required data separately from the included studies. Disagreements encountered during the process were resolved through discussion with the group. The following data were extracted from the final study: author, year, country, subject characteristics, intervention characteristics, and ADHD-related outcome indicators.

### 2.6. Quality assessment

The risk of bias was assessed using the Cochrane System Risk of Bias Assessment tool *via* Review Manager 5.4 software, which evaluates the studies’ quality on seven indicators: 1. Random sequence generation; 2. Allocation concealment; 3. Blinding of participants and personnel; 4. Blinding of outcome assessment; 5. Incomplete outcome data; 6. Selective reporting; and 7. Other bias.

### 2.7. Statistical analysis

We computed the standardized mean difference (SMD) and 95% CIs for continuous outcomes. The *P*-value of the chi-square test and the I2 index from the heterogeneity test were used to express the level of statistical heterogeneity. Different effect models were selected according to the level of heterogeneity of the test data. When the level of heterogeneity was low, a fixed-effects model (*P* ≥ 0.1 and I2 ≤ 50%) was used to analyze the data. Otherwise, a random-effects model (*P* < 0.1 or I2 values >50%) was used (Higgins et al., 2003).

According to the PRISMA NMA recommendations, we aggregated and analyzed NMA data using Markov chain Monte Carlo simulation chains in a Bayesian-based framework and Stata software (version 15.1) (Moher et al., 2015; Vats et al., 2019). We will employ the nodal method to quantify and demonstrate the congruence between indirect and direct comparisons, as obtained by Stata software instructions. If the *p*-value is greater than 0.05, the agreement test is passed.

Network meta-analysis was performed by employing a Bayesian model. The data were preprocessed using network group commands, and a mesh evidence map was drawn. The dots in the mesh evidence plot represent one intervention type, and the larger the area of its dots represents, the more significant the number of patients included in the study for the intervention. The line connecting the two dots is a direct comparison of the two interventions, and the thickness of the line represents the number of included studies. The larger the number of included studies, the thicker the line (Chaimani et al., 2013). The effects of the different movement methods were ranked. The effects of the different exercise modalities were ranked, the surface under the cumulative ranking curve (SUCRA) was obtained, and the probability ranking was plotted in a table. SUCRA is expressed as a percentage. The larger the percentage, the more effective the intervention. Additionally, to check for publication bias and minor sample study effects, we generated funnel plots for outcome indicators with study numbers >10 and used symmetry criteria to check (Khera et al., 2016). Stata15.1 was used to perform all statistical analyzes.

## 3. Results

### 3.1. Trial selection

**FIGURE 1:**
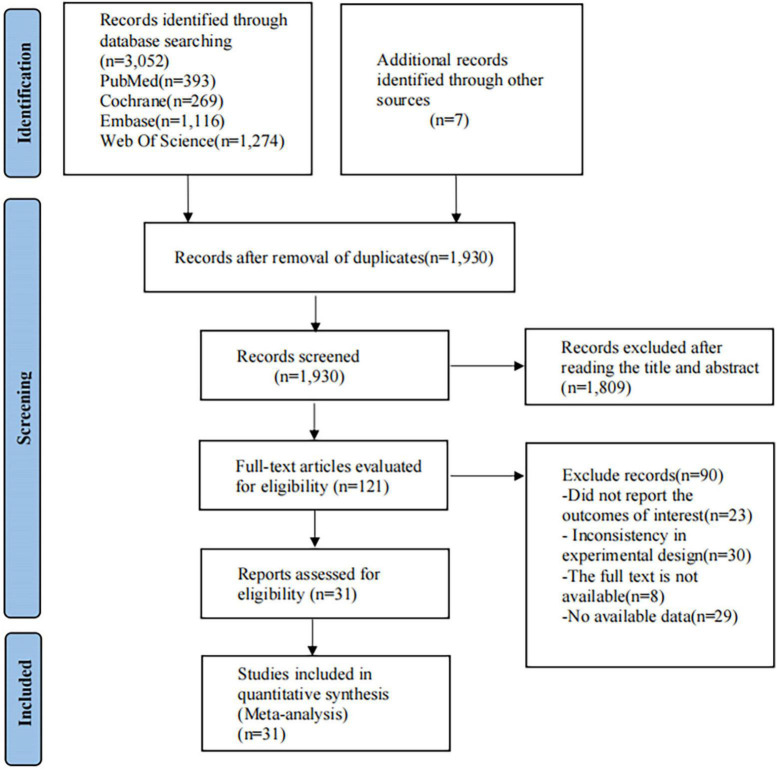
Preferred Reporting Items for Systematic Reviews and Meta-Analyzes flow diagram of the study process.

A total of 3,052 citations are yielded in the initial search of electronic databases, and an additional seven documents were manually searched. After removing duplicate studies (*n* = 1,129), 1,930 relevant papers remained. Subsequently, through screening, 1,809 papers were removed, and 121 papers suitable for full-text review remained, of which 90 were further eliminated. Finally, 31 studies were adopted for quantitative synthesis (Figure 1).

### 3.2. Trial characteristics

Characteristics of studies adopted are shown in Table 1, all of which were published between 2004 and 2022. The country with the highest number of included studies was Iran, with a total number of five papers. The sample size ranged from 5 to 104 for the experimental group and 5–98 for the control group, with relatively more men in the included studies. The included experimental and control groups’ mean age was less than or equal to 16 years. Interventions included cognitive-motor training (6 studies) (Hoza et al., 2015; Pan et al., 2016; Meßler et al., 2018; Benzing and Schmidt, 2019; Kadri et al., 2019; Mansson et al., 2019), combination exercise (6 studies) (Kang et al., 2011; Choi et al., 2015; Ziereis and Jansen, 2015; Bustamante et al., 2016; Memarmoghaddam et al., 2016; Lee et al., 2017), traditional aerobic exercise (5 studies) (Ahmed and Mohamed, 2011; Hoza et al., 2015; Gelade et al., 2017; Meßler et al., 2018; Kadri et al., 2019), acute aerobic exercise (4 studies) (Chang et al., 2012; Gawrilow et al., 2016; Ludyga et al., 2017; Benzing et al., 2018), aquatic exercise (3 studies) (Chang et al., 2014; Da Silva et al., 2020; Sabzi et al., 2021), horsemanship (3 studies) (García-Gómez et al., 2016; Oh et al., 2018; Ahn et al., 2021), perceptual-motor training (3 studies) (Yazd et al., 2015; Hattabi et al., 2019; Sani et al., 2022), mind-body exercise (2 studies) (Jensen and Kenny, 2004; Milligan et al., 2019), as well as sensory integration training (1 study) (Faramarzi et al., 2016). The outcome indicators for ADHD consisted of motor ability, social problems, attention problems, cognitive flexibility, inhibition switching, as well as the working memory.

**TABLE 1:** Summary table of included reviews.

Study	Country	Sample size		Gender (M/F)	Mean age (year)		Intervention						Outcome	ADHD diagnostics
		**EG**	**CG**		**EG**	**CG**	**EG**			**CG**				
							**Intervention content**	**Intervention time, frequency, period**	**Tape**	**Intervention content**	**Interven- tion time, frequency, period**	**Tape**		
Oh et al. (2018)	Korea	17	17	31/3	8.30	8.00	Horsemanship practice	60 min, 2 weekly, 12 weeks	HMS	Pharmacotherapy	Consistent with EG	PCT	A1;A2	DSM-4
Meßler et al. (2018)	Germany	14	14	28/0	11	11	High intensity interval training	25 min, 3 weekly, 3 weeks	TAE	Low-to-moderate intensity ball games	60 min, 3 weekly 3 weeks	CMT	A1;A2;A3	DSM-4
Sabzi et al. (2021)	Iran	23	23	23/23	9.60	9.50	Water treadmill exercise	30 min, 3 weekly, 8 weeks	AE	NI	NI	NI	A3	DSM-5
Mansson et al. (2019)	Denmark	64	64	109/19	11.39	11.63	Target-shooting sport	20–45 min, 1 weekly, 24 weeks	CMT	NI	NI	NI	A5	DSM-4
Sani et al. (2022)	Iran	20	20	29/11	7.50	7.78	Perceptual-motor training	40–45 min, 3 weekly, 7 weeks	PMT	Neurofeedback training	Consistent with EG	NFT	A1;A5	DSM–5
Yazd et al. (2015)	Iran	12	12	20/4	8.7	8.00	Perceptual-motor training	NR min, 3 weekly, 6 years	PMT	Pharmacotherapy	NR min 3 weekly 6 years	PCT	A1	DSM-4
Ahn et al. (2021)	Korea	8	7	12/3	7.50	7.14	Horsemanship practice	40 min, 2 weekly, 16 weeks	HMS	NI	NI	NI	A2;A3	DSM-5
Smith et al. (2020)	America	42	38	53/27	7.6	7.20	Computerized cognitive training + physical exercises	120 min, 4 weekly, 15 weeks	CMT	Pharmacotherapy	NR	PCT	A5;A6	DSM-4
Da Silva et al. (2020)	Brazil	10	10	14/6	12	12.00	Swimming training	45 min, 2 weekly, 8 weeks	AE	NI	NI	NI	A1;A2;A4	DSM-4
Milligan et al. (2019)	Canada	48	38	72/14	13.10	12.82	Mindfulness martial arts	90 min, 1 weekly, 20 weeks	MBE	Mental health treatments+ educational interventions	NR	CI	A2;A4;A5;A6	DSM-4
Chang et al. (2012)	China	20	20	37/3	10.42	10.45	Aerobic exercise	30 min, 1 weekly, 1 week	AAE	Watch video	Consistent with EG	CI	A4;A5	DSM-4
Chang et al. (2014)	China	14	13	23/4	8.19	8.78	Water exercise	90 min, 2 weekly, 8 weeks	AE	NI	NI	NI	A1;A4;A5	DSM-4
Kang et al. (2011)	Korea	15	13	28/0	8.4	8.6	Sports therapy	90 min, 2 weekly, 6 weeks	CE	Education for behavior	NR	CI	A2;A5;A6	DSM-4
Gawrilow et al. (2016)	Germany	23	24	47/0	10.47	10.47	Trampoline	5 min, 1 weekly, 1 week	AAE	Sedentary task	Consistent with EG	CI	A4;A5	ICD-10
Choi et al. (2015)	Korea	13	17	30/0	15.80	16.00	Sports therapy	90 min, 3 weekly, 6 weeks	CE	Watch video	Consistent with EG	CI	A4;A5	DSM-4
Ziereis and Jansen (2015)	Germany	13	16	21/8	9.2	9.5	Sports therapy	60 min, 1 weekly, 12 weeks	CE	NI	NI	NI	A1;A4;A6	ICD-10
Bustamante et al. (2016)	America	18	16	24/10	9.40	8.70	Physically active games	90 min, 5 weekly, 10 weeks	CE	Sedentary task	Consistent with EG	CI	A2;A5;A6	DSM-4
Memarmoghaddam et al. (2016)	Iran	19	17	NR	8.31	8.29	Selected exercise program	90 min, 3 weekly, 8 weeks	CE	NI	NI	NI	A4;A5	SNAP-4
Pan et al. (2016)	China	16	16	32/0	8.93	8.87	Table tennis exercise	70 min, 2 weekly, 12 weeks	CMT	NI	NI	NI	A2;A3;A4	DSM-4
Lee et al. (2017)	Korea	6	6	12/0	10.46	10.50	Combined exercise program	60 min, 3 weekly, 12 weeks	CE	NI	NI	NI	A4	DSM-4
Benzing et al. (2018)	Switzerland	24	22	38/8	10.46	10.50	Exergame (sports games)	15 min, 1 weekly, 1 week	AAE	Watch video	Consistent with EG	CI	A4;A5;A6	ICD-10
Benzing and Schmidt (2019)	Switzerland	28	23	43/8	10.46	10.39	Exergame for physical and cognitive challenges	30 min, 3 weekly, 8 weeks	CMT	Watch video	Consistent with EG	CI	A5;A6	ICD-10
Kadri et al. (2019)	Tunisia	20	20	36/4	14.5	14.20	Taekwondo	30 min, 2 weekly, 1.5 years	CMT	Traditional physical education classes	Consistent with EG	TAE	A2;A4;A5;A6	DSM-4
Gelade et al. (2017)	Netherlands	39	CG1:36 CG2:37	85/27	9.96	CG1:9.11 CG2:9.80	Neurofeedback (theta/beta training)	20 min, 3 weekly, 10–12 weeks	NFB	CG1: methylphenidate CG2: aerobic exercise	CG1:NR CG2:20 min 3 weekly, 10–12 weeks	CG1: PCT CG2: TAE	A2;A5;A6	DSM-4
Faramarzi et al. (2016)	Iran	10	10	20/0	Elementary students	Elementary students	Sensory integration training	45 min, 2 weekly, 6 weeks	SIT	NI	NI	NI	A5	Conner’s rating scale
Ludyga et al. (2017)	Switzerland	5	8	NR	12.80	13.50	Aerobic exercise	20 min, 1 weekly, 1 week	AAE	Watch video	Consistent with EG	CI	A5	DSM-4
Ahmed and Mohamed (2011)	Egypt	42	42	54/30	13.90	13.80	Aerobic exercises program	40–50 min, 3 weekly, 10 weeks	TAE	NI	NI	NI	A1;A2	DSM-4
García-Gómez et al. (2016)	Spain	9	5	10/4	10.65	10.20	Equestrian therapy	45 min, 2 weekly, 12 weeks	HMS	NI	NI	NI	A5;A6	DSM-4
Hattabi et al. (2019)	Tunisia	20	20	5/35	9.95	9.75	Perceptual motor water exercise	90 min, 3 weekly, 12 weeks	PMT	NI	NI	NI	A1;A5;A6	Conner’s rating scale
Hoza et al. (2015)	America	104	98	108/94	6.83	6.83	Aerobic physical activity	31 min, 5 weekly, 12 weeks	TAE	Sedentary task	Consistent with EG	CI	A2	DSM-4
Jensen and Kenny (2004)	Australia	11	8	19/0	10.63	9.35	Yoga	60 min, 1 weekly, 20 weeks	MBE	Cooperative games	Consistent with EG	CI	A2;A4	DSM-4

ADHD, attention-deficit/hyperactivity disorder; EG, experimental group; CG, control group; NR, no report; NI, no intervention; HMS, horsemanship; CBE, combination exercise; PMT, perceptual-motor training; CMT, cognitive-motor training; AE, aquatic exercise; MBE, mind-body exercise; AAE, acute aerobic exercise; TAE, traditional aerobic exercise; SIT, sensory integration training; NFB, neurofeedback; CI, cognitive intervention; PCT, pharmacotherapy; NIMH DISC-IV, national institute of mental health diagnostic interview schedule for children version IV; DSM-4 and DSM-5, diagnostic and statistical manual of mental disorders, fourth edition and fifth edition; ICD-10, international classification of diseases, tenth revision; SNAP-4, Swanson, Nolan and Pelham rating scale, fourth edition; A1, motor ability; A2, attention problems; A3, social problems; A4, cognitive flexibility; A5, inhibition switching; A6, working memory.

### 3.3. Risk of bias

**FIGURE 2:**
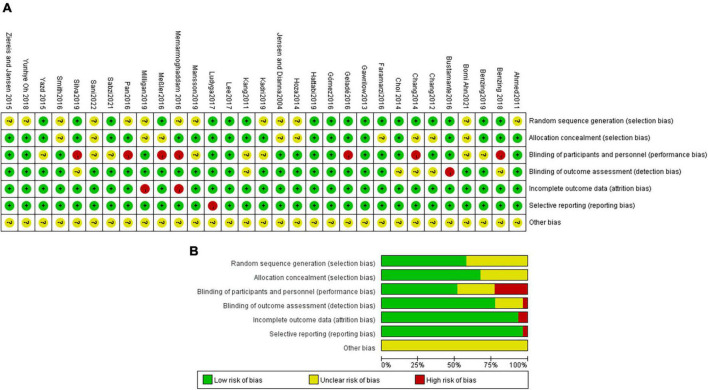
**(A)** Methodological quality of included studies. **(B)** The distribution of the methodological quality of included studies.

Eighteen studies (58.1%) had a low risk of bias with respect to random sequence generation. Twenty-one studies (67.7%) had a low risk of bias with respect to allocation concealment. Sixteen studies (51.6%) had a low risk of bias with respect to the blinding of participants and personnel. Twenty-four studies (77.4%) had a low risk of bias with respect to the blinding of outcome assessments. Twenty-nine studies (93.5%) had a low risk of bias with respect to incomplete outcome data. Thirty studies (96.8%) had a low risk of bias with respect to selective reporting. Other biases are not known. Details of the evaluation of bias results for the included literature are shown in Figures 2A, B.

### 3.4. Network meta-analysis

The complete NMA figure will be presented in Supplementary Appendixes B1–6.

#### 3.4.1. Motor ability

Seven studies (Chang et al., 2014; Yazd et al., 2015; Pan et al., 2016; Meßler et al., 2018; Oh et al., 2018; Da Silva et al., 2020; Sani et al., 2022) reported on the motor ability of children with ADHD, and a total of nine interventions are involved. As shown in Table 2, the statistically significant results of the network meta-analysis were as follows: Perceptual-motor training [*MD* = 5.26, 95% *CI* = (1.14, 9.38)], traditional aerobic exercise [*MD* = 4.74, 95% *CI* = (0.31, 9.16)], and aquatic exercise [*MD* = 3.66, 95% *CI* = (0.73, 6.60)], which were more effective than that with no intervention. Compared with pharmacotherapy, perceptual-motor training [*MD* = 9.76, 95% *CI* = (4.92, 14.60)], traditional aerobic exercise [*MD* = 9.23, 95% *CI* = (1.76, 16.70)], aquatic exercise [*MD* = 8.16, 95% *CI* = (1.18, 15.15)], combination exercise [*MD* = 7.38, 95% *CI* = (0.17, 14.59)] were more effective. Compared with neurofeedback, perceptual-motor training [*MD* = 28.68, 95% *CI* = (18.20, 39.15)], traditional aerobic exercise [*MD* = 28.15, 95% *CI* = (16.07, 40.23)], aquatic exercise [*MD* = 27.08, 95% *CI* = (15.46, 38.70)], combination exercise [*MD* = 26.30, 95% *CI* = (14.54, 38.05)], cognitive-motor training [*MD* = 26.11, 95% *CI* = (13.27, 38.96)], and horsemanship [*MD* = 24.91, 95% *CI* = (11.12, 38.70)] were more effective. In SUCRA, perceptual-motor training ranked first in terms of the probability of the effect of different interventions on motor performance (SUCRA: 82.7%, as shown in Supplementary Appendix C1).

**TABLE 2:** League table on motor ability.

PMT	TAE	AE	CBE	CMT	HMS	NI	PCT	NFB
PMT	−0.53 (−6.56, 5.50)	−1.60 (−6.65, 3.46)	−2.38 (−7.72, 2.96)	−2.57 (−10.02, 4.89)	−3.77 (−12.74, 5.20)	−5.26 (−9.38, 1.14)	−9.76 (−14.60, −4.92)	−28.68 (−39.15, −18.20)
0.53 (−5.50, 6.56)	TAE	−1.07 (−6.34, 4.20)	−1.85 (−7.43, 3.72)	−2.04 (−6.45, 2.37)	−3.24 (−13.86, 7.38)	−4.74 (−9.16, −0.31)	−9.23 (−16.70, −1.76)	−28.15 (−40.23, −16.07)
1.60 (−3.46, 6.65)	1.07 (−4.20, 6.34)	AE	−0.78 (−5.27, 3.71)	−0.97 (−7.81, 5.87)	−2.17 (−12.45, 8.11)	−3.66 (−6.60, −0.73)	−8.16 (−15.15, −1.18)	−27.08 (−38.70, −15.46)
2.38 (−2.96, 7.72)	1.85 (−3.72, 7.43)	0.78 (−3.71, 5.27)	CBE	−0.18 (−7.29, 6.92)	−1.39 (−11.82, 9.05)	−2.88 (−6.28, 0.52)	−7.38 (−14.59, −0.17)	−26.30 (−38.05, −14.54)
2.57 (−4.89, 10.02)	2.04 (−2.37, 6.45)	0.97 (−5.87, 7.81)	0.18 (−6.92, 7.29)	CMT	−1.20 (−12.51, 10.10)	−2.70 (−8.93, 3.54)	−7.19 (−15.61, 1.22)	−26.11 (−38.96, −13.27)
3.77 (−5.20, 12.74)	3.24 (−7.38, 13.86)	2.17 (−8.11, 12.45)	1.39 (−9.05, 11.82)	1.20 (−10.10, 12.51)	HMS	−1.50 (−11.36, 8.37)	−5.99 (−13.55, 1.56)	−24.91 (−38.70, −11.12)
**5.26 (1.14, 9.38)**	**4.74 (0.31, 9.16)**	**3.66 (0.73, 6.60)**	2.88 (−0.52, 6.28)	2.70 (−3.54, 8.93)	1.50 (−8.37, 11.36)	NI	−4.50 (−10.85, 1.86)	−23.42 (−34.67, −12.16)
**9.76 (4.92, 14.60)**	**9.23 (1.76, 16.70)**	**8.16 (1.18, 15.15)**	**7.38 (0.17, 14.59)**	7.19 (−1.22, 15.61)	5.99 (−1.56, 13.55)	4.50 (−1.86, 10.85)	PCT	−18.92 (−30.45, −7.38)
**28.68 (18.20, 39.15)**	**28.15 (16.07, 40.23)**	**27.08 (15.46, 38.70)**	**26.30 (14.54, 38.05)**	**26.11 (13.27, 38.96)**	**24.91 (11.12, 38.70)**	**23.42 (12.16, 34.67)**	**18.92 (7.38, 30.45)**	NFB

The bold values represent the signify statistical significance.

#### 3.4.2. Attention problems

Fourteen studies (Jensen and Kenny, 2004; Ahmed and Mohamed, 2011; Kang et al., 2011; Hoza et al., 2015; Bustamante et al., 2016; García-Gómez et al., 2016; Janssen et al., 2016; Pan et al., 2016; Meßler et al., 2018; Oh et al., 2018; Kadri et al., 2019; Milligan et al., 2019; Da Silva et al., 2020; Ahn et al., 2021) reported on the attention problems of children with ADHD, and a total of ten interventions are involved. As shown in Table 3, there is no statistical significance for each intervention in the network meta-analysis results. In SUCRA, aquatic exercise ranked first in terms of the probability of the effect of different interventions on the reduction of attention problems (SUCRA: 80.9%, as shown in Supplementary Appendix C2).

**TABLE 3:** League table on attention problems.

AE	PCT	CMT	HMS	MBE	NI	CBE	NFB	CI	TAE
AE	4.71 (−35.80, 45.21)	8.63 (−24.77, 42.03)	9.97 (−23.99, 43.92)	19.34 (−22.39, 61.07)	18.81 (−8.53, 46.16)	21.55 (−19.94, 63.04)	24.65 (−31.56, 80.87)	25.21 (−11.07, 61.48)	26.82 (−5.04, 58.68)
−4.71 (−45.21, 35.80)	PCT	3.92 (−31.05, 38.89)	5.26 (−21.49, 32.01)	14.63 (−27.98, 57.24)	14.11 (−16.82, 45.03)	16.84 (−26.25, 59.94)	19.95 (−27.85, 67.74)	20.50 (−17.47, 58.47)	22.11 (−10.69, 54.92)
−8.63 (−42.03, 24.77)	−3.92 (−38.89, 31.05)	CMT	1.34 (−27.84, 30.51)	10.71 (−23.14, 44.56)	10.18 (−11.66, 32.03)	12.92 (−21.17, 47.01)	16.02 (−35.98, 68.03)	16.58 (−10.65, 43.81)	18.19 (−0.52, 36.90)
−9.97 (−43.92, 23.99)	−5.26 (−32.01, 21.49)	−1.34 (−30.51, 27.84)	HMS	9.37 (−29.70, 48.45)	8.85 (−12.40, 30.10)	11.58 (−27.74, 50.91)	14.68 (−35.98, 65.35)	15.24 (−18.38, 48.86)	16.85 (−10.79, 44.49)
−19.34 (−61.07, 22.39)	−14.63 (−57.24, 27.98)	−10.71 (−44.56, 23.14)	−9.37 (−48.45, 29.70)	MBE	−0.53 (−35.16, 34.11)	2.21 (−26.80, 31.22)	5.31 (−51.68, 62.30)	5.87 (−14.46, 26.19)	7.48 (−21.28, 36.24)
−18.81 (−46.16, 8.53)	−14.11 (−45.03, 16.82)	−10.18 (−32.03, 11.66)	−8.85 (−30.10, 12.40)	0.53 (−34.11, 35.16)	NI	2.74 (−32.04, 37.51)	5.84 (−44.80, 56.48)	6.39 (−21.77, 34.55)	8.00 (−12.63, 28.64)
−21.55 (−63.04, 19.94)	−16.84 (−59.94, 26.25)	−12.92 (−47.01, 21.17)	−11.58 (−50.91, 27.74)	−2.21 (−31.22, 26.80)	−2.74 (−37.51, 32.04)	CBE	3.10 (−54.17, 60.38)	3.66 (−17.09, 24.41)	5.27 (−23.63, 34.17)
−24.65 (−80.87, 31.56)	−19.95 (−67.74, 27.85)	−16.02 (−68.03, 35.98)	−14.68 (−65.35, 35.98)	−5.31 (−62.30, 51.68)	−5.84 (−56.48, 44.80)	−3.10 (−60.38, 54.17)	NFB	0.56 (−52.95, 54.06)	2.17 (−47.69, 52.02)
−25.21 (−61.48, 11.07)	−20.50 (−58.47, 17.47)	−16.58 (−43.81, 10.65)	−15.24 (−48.86, 18.38)	−5.87 (−26.19, 14.46)	−6.39 (−34.55, 21.77)	−3.66 (−24.41, 17.09)	−0.56 (−54.06, 52.95)	CI	1.61 (−18.67, 21.89)
−26.82 (−58.68, 5.04)	−22.11 (−54.92, 10.69)	−18.19 (−36.90, 0.52)	−16.85 (−44.49, 10.79)	−7.48 (−36.24, 21.28)	−8.00 (−28.64, 12.63)	−5.27 (−34.17, 23.63)	−2.17 (−52.02, 47.69)	−1.61 (−21.89, 18.67)	TAE

#### 3.4.3. Social problems

Five studies (Pan et al., 2016; Meßler et al., 2018; Oh et al., 2018; Ahn et al., 2021; Sabzi et al., 2021) reported on the social problems of children with ADHD, and a total of six interventions are involved. As shown in Table 4, the statistically significant results of the network meta-analysis were as follows: Aquatic exercise [*MD* = −3.70, 95% *CI* = (−5.03, −2.37)] was more effective than that with no intervention. In the SUCRA, aquatic exercise ranked first in terms of the probability of the effect of different interventions on the reduction of social problems (SUCRA: 79.4%, as shown in Supplementary Appendix C3).

**TABLE 4:** League table on social problems.

HMS	PCT	AE	TAE	CMT	NI
HMS	1.83 (−3.46, 7.12)	6.41 (−12.08, 24.91)	8.10 (−10.78, 26.98)	8.48 (−10.39, 27.35)	10.11 (−8.34, 28.57)
−1.83 (−7.12, 3.46)	PCT	4.58 (−14.65, 23.82)	6.27 (−13.33, 25.88)	6.65 (−12.95, 26.25)	8.28 (−10.91, 27.48)
−6.41 (−24.91, 12.08)	−4.58 (−23.82, 14.65)	AE	1.69 (−2.53, 5.90)	2.07 (−2.11, 6.25)	3.70 (2.37, 5.03)
−8.10 (−26.98, 10.78)	−6.27 (−25.88, 13.33)	−1.69 (−5.90, 2.53)	TAE	0.38 (−0.13, 0.89)	2.01 (−1.99, 6.01)
−8.48 (−27.35, 10.39)	−6.65 (−26.25, 12.95)	−2.07 (−6.25, 2.11)	−0.38 (−0.89, 0.13)	CMT	1.63 (−2.33, 5.60)
−10.11 (−28.57, 8.34)	−8.28 (−27.48, 10.91)	−**3.70 (**−**5.03,**−**2.37)**	−2.01 (−6.01, 1.99)	−1.63 (−5.60, 2.33)	NI

The bold values represent the signify statistical significance.

#### 3.4.4. Cognitive flexibility

Fourteen studies (Jensen and Kenny, 2004; Kang et al., 2011; Chang et al., 2012, 2014; Hoza et al., 2015; Ziereis and Jansen, 2015; Gawrilow et al., 2016; Memarmoghaddam et al., 2016; Pan et al., 2016; Lee et al., 2017; Benzing et al., 2018; Kadri et al., 2019; Milligan et al., 2019; Da Silva et al., 2020) reported on the cognitive flexibility of children with ADHD, and a total of nine interventions are involved. As shown in Table 5, the statistically significant results of the network meta-analysis were as follows: Aquatic exercise [*MD* = 19.65, 95% *CI* = (3.91, 35.40)] was more effective than combination exercise. Aquatic exercise [*MD* = 22.16, 95% *CI* = (8.36, 35.97)] was more effective than that with no intervention. Compared to traditional aerobic exercise, aquatic exercise [*MD* = 43.47, 95% *CI* = (17.05, 69.89)], acute aerobic exercise [*MD* = 32.10, 95% *CI* = (1.38, 62.82)], and cognitive-motor training [*MD* = 30.06, 95% *CI* = (16.23, 43.89)] were more effective. In SUCRA, aquatic exercise ranked first in terms of the probability of the effect of different interventions on cognitive flexibility (SUCRA: 86.6%, as shown in Supplementary Appendix C4).

**TABLE 5:** League table on cognitive flexibility.

AE	MBE	CI	AAE	CMT	PCT	CBE	NI	TAE
AE	−8.89 (−43.34, 25.57)	−10.17 (−42.15, 21.80)	−11.37 (−36.93, 14.19)	−13.41 (−35.97, 9.15)	−14.24 (−43.18, 14.70)	−19.65 (−35.40, −3.91)	−22.16 (−35.97, −8.36)	−43.47 (−69.89, −17.05)
8.89 (−25.57, 43.34)	MBE	−1.28 (−14.23, 11.66)	−2.48 (−25.53, 20.56)	−4.52 (−40.47, 31.42)	−5.36 (−45.57, 34.85)	−10.77 (−43.23, 21.70)	−13.27 (−44.90, 18.35)	−34.58 (−72.95, 3.78)
10.17 (−21.80, 42.15)	1.28 (−11.66, 14.23)	CU	−1.20 (−20.34, 17.94)	−3.24 (−36.81, 30.33)	−4.07 (−42.18, 34.04)	−9.48 (−39.30, 20.34)	−11.99 (−40.89, 16.91)	−33.30 (−69.45, 2.86)
11.37 (−14.19, 36.93)	2.48 (−20.56, 25.53)	1.20 (−17.94, 20.34)	AAE	−2.04 (−29.60, 25.51)	−2.87 (−35.80, 30.05)	−8.28 (−31.11, 14.54)	−10.79 (−32.42, 10.84)	−32.10 (−62.82, −1.38)
13.41 (−9.15, 35.97)	4.52 (−31.42, 40.47)	3.24 (−30.33, 36.81)	2.04 (−25.51, 29.60)	CMT	−0.83 (−19.03, 17.37)	−6.24 (−25.63, 13.15)	−8.75 (−26.60, 9.10)	−30.06 (−43.89, −16.23)
14.24 (−14.70, 43.18)	5.36 (−34.85, 45.57)	4.07 (−34.04, 42.18)	2.87 (−30.05, 35.80)	0.83 (−17.37, 19.03)	PCT	−5.41 (−31.95, 21.13)	−7.92 (−33.36, 17.52)	−29.23 (−52.08, −6.37)
**19.65 (3.91, 35.40)**	10.77 (−21.70, 43.23)	9.48 (−20.34, 39.30)	8.28 (−14.54, 31.11)	6.24 (−13.15, 25.63)	5.41 (−21.13, 31.95)	CBE	−2.51 (−10.09, 5.07)	−23.82 (−47.58, −0.05)
**22.16 (8.36, 35.97)**	13.27 (−18.35, 44.90)	11.99 (−16.91, 40.89)	10.79 (−10.84, 32.42)	8.75 (−9.10, 26.60)	7.92 (−17.52, 33.36)	2.51 (−5.07, 10.09)	NI	−21.31 (−43.83, 1.21)
**43.47 (17.05, 69.89)**	34.58 (−3.78, 72.95)	33.30 (−2.86, 69.45)	**32.10 (1.38, 62.82)**	**30.06 (16.23, 43.89)**	**29.23 (6.37, 52.08)**	**23.82 (0.05, 47.58)**	21.31 (−1.21,43.83)	TAE

The bold values represent the signify statistical significance.

#### 3.4.5. Inhibition switching

Eighteen studies (Kang et al., 2011; Chang et al., 2012, 2014; Choi et al., 2015; Hoza et al., 2015; Bustamante et al., 2016; Faramarzi et al., 2016; García-Gómez et al., 2016; Gawrilow et al., 2016; Janssen et al., 2016; Memarmoghaddam et al., 2016; Ludyga et al., 2017; Benzing et al., 2018; Benzing and Schmidt, 2019; Hattabi et al., 2019; Kadri et al., 2019; Mansson et al., 2019; Milligan et al., 2019; Sani et al., 2022) reported the inhibition switching of children with ADHD, and a total of 13 interventions are involved. As shown in Table 6, the statistically significant results of the network meta-analysis were as follows: Cognitive-motor training [*MD* = −67.14, 95% *CI* = (−130.91, −3.37)] was more effective than cognitive intervention. Compared with aquatic exercise, cognitive-motor training [*MD* = −146.75, 95% *CI* = (−257.37, 36.13)], perceptual-motor training [*MD* = −143.08, 95% *CI* = (−262.96, 23.19)], combination exercise [*MD* = −129.35, 95% *CI* = (−232.92, 25.77)], and acute aerobic exercise [*MD* = −112.14, 95% *CI* = (−214.20, 10.09)] were more effective. In SUCRA, cognitive-motor training ranked first in the probability of the effect of different interventions on inhibition switching (SUCRA: 83.5%, as shown in Supplementary Appendix C5).

**TABLE 6:** League table on inhibition switching.

CMT	PMT	CBE	NFB	AAE	TAE	MBE	PCT	SIT	HMS	NI	CI	AE
CMT	3.67 (−86.92, 94.26)	17.40 (−56.88, 91.68)	31.40 (−57.04, 119.83)	34.61 (−27.75, 96.96)	44.27 (−29.38, 117.91)	46.46 (−66.93, 159.84)	46.34 (−33.15, 125.82)	54.59 (−54.83, 164.00)	55.91 (−53.49, 165.31)	60.86 (−3.10, 124.82)	67.14 (3.37, 130.91)	146.75 (36.13, 257.37)
−3.67 (−94.26, 86.92)	PMT	13.73 (−76.62, 104.09)	27.73 (−56.77, 112.23)	30.94 (−53.19, 115.06)	40.60 (−61.56, 142.76)	42.79 (−86.99, 172.56)	42.67 (−62.63, 147.96)	50.92 (−67.83, 169.66)	52.24 (−66.49, 170.97)	57.19 (−21.00, 135.38)	63.47 (−26.71, 153.65)	143.08 (23.19, 262.96)
−17.40 (−91.68, 56.88)	−13.73 (−104.09, 76.62)	CBE	13.99 (−83.90, 111.89)	17.20 (−42.57, 76.98)	26.86 (−69.39, 123.12)	29.05 (−83.68, 141.79)	28.93 (−71.39, 129.26)	37.18 (−65.44, 139.81)	38.51 (−64.12, 141.13)	43.46 (−7.17, 94.08)	49.74 (−13.24, 112.71)	129.35 (25.77, 232.92)
−31.40 (−119.83, 57.04)	−27.73 (−112.23, 56.77)	−13.99 (−111.89, 83.90)	NFB	3.21 (−85.40, 91.82)	12.87 (−76.97, 102.71)	15.06 (−117.29, 147.41)	14.94 (−77.44, 107.32)	23.19 (−102.51, 148.89)	24.51 (−101.17, 150.20)	29.46 (−59.43, 118.36)	35.74 (−58.02, 129.50)	115.35 (−11.46, 242.17)
−34.61 (−96.96, 27.75)	−30.94 (−115.06, 53.19)	−17.20 (−76.98, 42.57)	−3.21 (−91.82, 85.40)	AAE	9.66 (−76.47, 95.79)	11.85 (−91.48, 115.18)	11.73 (−78.85, 102.31)	19.98 (−80.77, 120.72)	21.30 (−79.43, 122.03)	26.25 (−23.46, 75.96)	32.53 (−12.79, 77.85)	112.14 (10.09, 214.20)
−44.27 (−117.91, 29.38)	−40.60 (−142.76, 61.56)	−26.86 (−123.12, 69.39)	−12.87 (−102.71, 76.97)	−9.66 (−95.79, 76.47)	TAE	2.19 (−127.24, 131.62)	2.07 (−84.58, 88.71)	10.32 (−114.78, 135.42)	11.64 (−113.44, 136.73)	16.59 (−71.61, 104.79)	22.87 (−66.54, 112.28)	102.48 (−23.74, 228.71)
−46.46 (−159.84, 66.93)	−42.79 (−172.56, 86.99)	−29.05 (−141.79, 83.68)	−15.06 (−147.41, 117.29)	−11.85 (−115.18, 91.48)	−2.19 (−131.62, 127.24)	MBE	−0.12 (−132.62, 132.38)	8.13 (−133.02, 149.27)	9.45 (−131.68, 150.59)	14.40 (−95.82, 124.62)	20.68 (−74.26, 115.62)	100.29 (−41.87, ‘242.45)
−46.34 (−125.82, 33.15)	−42.67 (−147.96, 62.63)	−28.93 (−129.26, 71.39)	−14.94 (−107.32, 77.44)	−11.73 (−102.31,7 8.85)	−2.07 (−88.71, 84.58)	0.12 (−132.38, 132.62)	PCT	8.25 (−119.96, 136.45)	9.57 (−118.62, 137.76)	14.52 (−78.01, 107.05)	20.80 (−72.98, 114.59)	100.41 (−28.93, 229.76)
−54.59 (−164.00, 54.83)	−50.92 (−169.66, 67.83)	−37.18 (−139.81, 65.44)	−23.19 (−148.89, 102.51)	−19.98 (−120.72, 80.77)	−10.32 (−135.42, 114.78)	−8.13 (−149.27, 133.02)	−8.25 (−136.45, 119.96)	SIT	1.32 (−125.96, 128.60)	6.27 (−83.86,96.41)	12.55 (−93.37, 118.48)	92.16 (−36.14, 220.47)
−55.91 (−165.31, 53.49)	−52.24 (−170.97, 66.49)	−38.51 (−141.13, 64.12)	−24.51 (−150.20, 101.17)	−21.30 (−122.03, 79.43)	−11.64 (−136.73, 113.44)	−9.45 (−150.59, 131.68)	−9.57 (−137.76, 118.62)	−1.32 (−128.60, 125.96)	HMS	4.95 (−85.17, 95.07)	11.23 (−94.67, 117.13)	90.84 (−37.47, 219.15)
−60.86 (−124.82, 3.10)	−57.19 (−135.38, 21.00)	−43.46 (−94.08, 7.17)	−29.46 (−118.36, 59.43)	−26.25 (−75.96, 23.46)	−16.59 (−104.79, 71.61)	−14.40 (−124.62, 95.82)	−14.52 (−107.05, 78.01)	−6.27 (−96.41, 83.86)	−4.95 (−95.07, 85.17)	NI	6.28 (−52.26, 64.82)	85.89 (−5.83, 177.62)
**−67.14 (−130.91,** **−3.37)**	−63.47 (−153.65, 26.71)	−49.74 (−112.71, 13.24)	−35.74 (−129.50, 58.02)	−32.53 (−77.85, 12.79)	−22.87 (−112.28, 66.54)	−20.68 (−115.62, 74.26)	−20.80 (−114.59, 72.98)	−12.55 (−118.48, 93.37)	−11.23 (−117.13, 94.67)	−6.28 (−64.82, 52.26)	CI	79.61 (−27.84, 187.06)
−**146.75 (−257.37,** **−36.13)**	**−143.08 (−262.96,** **−23.19)**	**−129.35 (−232.92,** **−25.77)**	−115.35 (−242.17, 11.46)	**−112.14 (−214.20,** **−10.09)**	−102.48 (−228.71, 23.74)	−100.29 (−242.45, 41.87)	−100.41 (−229.76, 28.93)	−92.16 (−220.47, 36.14)	−90.84 (−219.15, 37.47)	−85.89 (−177.62, 5.83)	−79.61 (−187.06, 27.84)	AE

The bold values represent the signify statistical significance.

#### 3.4.6. Working memory

Ten studies (Kang et al., 2011; Hoza et al., 2015; Ziereis and Jansen, 2015; Bustamante et al., 2016; Janssen et al., 2016; Benzing et al., 2018; Benzing and Schmidt, 2019; Hattabi et al., 2019; Kadri et al., 2019; Milligan et al., 2019) reported on the working memory of children with ADHD, and a total of nine interventions are involved. As shown in Table 7, the statistically significant results of the network meta-analysis were as follows: cognitive-motor training [*MD* = 9.45, 95% *CI* = (2.39, 16.51)] was more effective than traditional aerobic exercise. In SUCRA, perceptual-motor training ranked first in terms of the probability of effectiveness of different interventions on working memory (SUCRA: 73.3%, as shown in Supplementary Appendix C6).

**TABLE 7:** League table on working memory.

PMT	MBE	HMS	CMT	CBE	AAE	CI	NI	PCT	NFB	TAE
PMT	−1.18 (−21.98, 19.62)	−1.57 (−14.75, 11.60)	−3.71 (−21.19, 13.78)	−3.24 (−16.43, 9.95)	−4.83 (−22.95, 13.30)	−4.82 (−19.54, 9.90)	−4.85 (−14.15, 4.45)	−8.59 (−26.80, 9.61)	−10.24 (−29.38, 8.90)	−13.16 (−31.95, 5.64)
1.18 (−19.62, 21.98)	MBE	−0.39 (−21.20, 20.42)	−2.53 (−19.99, 14.94)	−2.06 (−18.16, 14.04)	−3.64 (−21.82, 14.53)	−3.64 (−18.33, 11.05)	−3.67 (−22.28, 14.94)	−7.41 (−25.62, 10.79)	−9.06 (−28.22, 10.09)	−11.98 (−30.80, 6.85)
1.57 (−11.60, 14.75)	0.39 (−20.42, 21.20)	HMS	−2.13 (−19.63, 15.36)	−1.67 (−14.88, 11.54)	−3.25 (−21.39, 14.89)	−3.25 (−17.98, 11.49)	−3.28 (−12.61, 6.05)	−7.02 (−25.24, 11.20)	−8.67 (−27.82, 10.48)	−11.58 (−30.39, 7.22)
3.71 (−13.78, 21.19)	2.53 (−14.94, 19.99)	2.13 (−15.36, 19.63)	CMT	0.47 (−11.03, 11.96)	−1.12 (−15.27, 13.03)	−1.11 (−10.55, 8.32)	−1.14 (−15.95, 13.67)	−4.89 (−10.07, 0.30)	−6.54 (−14.44, 1.37)	−9.45 (−16.51, −2.39)
3.24 (−9.95, 16.43)	2.06 (−14.04, 18.16)	1.67 (−11.54, 14.88)	−0.47 (−11.96, 11.03)	CBE	−1.58 (−14.10, 10.93)	−1.58 (−8.15, 4.99)	−1.61 (−10.97, 7.75)	−5.35 (−17.92, 7.21)	−7.00 (−20.89, 6.89)	−9.92 (−23.32, 3.49)
4.83 (−13.30, 22.95)	3.64 (−14.53, 21.82)	3.25 (−14.89, 21.39)	1.12 (−13.03, 15.27)	1.58 (−10.93, 14.10)	AAE	0.00 (−10.70, 10.71)	−0.02 (−15.60, 15.55)	−3.77 (−18.80, 11.27)	−5.42 (−21.58, 10.75)	−8.33 (−24.10, 7.44)
4.82 (−9.90, 19.54)	3.64 (−11.05, 18.33)	3.25 (−11.49, 17.98)	1.11 (−8.32, 10.55)	1.58 (−4.99, 8.15)	−0.00 (−10.71, 10.70)	CI	−0.03 (−11.45, 11.39)	−3.77 (−14.52, 6.97)	−5.42 (−17.71, 6.86)	−8.34 (−20.10, 3.42)
4.85 (−4.45, 14.15)	3.67 (−14.94, 22.28)	3.28 (−6.05, 12.61)	1.14 (−13.67, 15.95)	1.61 (−7.75, 10.97)	0.02 (−15.55, 15.60)	0.03 (−11.39, 11.45)	NI	−3.74 (−19.40, 11.91)	−5.39 (−22.13, 11.34)	−8.31 (−24.64, 8.03)
8.59 (−9.61, 26.80)	7.41 (−10.79, 25.62)	7.02 (−11.20, 25.24)	4.89 (−0.30, 10.07)	5.35 (−7.21, 17.92)	3.77 (−11.27, 18.80)	3.77 (−6.97, 14.52)	3.74 (−11.91, 19.40)	PCT	1.65 (−8.12, 4.82)	−4.56 (−10.51, 1.38)
10.24 (−8.90, 29.38)	9.06 (−10.09, 28.22)	8.67 (−10.48, 27.82)	6.54 (−1.37, 14.44)	7.00 (−6.89, 20.89)	5.42 (−10.75, 21.58)	5.42 (−6.86, 17.71)	5.39 (−11.34, 22.13)	1.65 (−4.82, 8.12)	NFB	−2.91 (−9.39, 3.56)
13.16 (−5.64, 31.95)	11.98 (−6.85, 30.80)	11.58 (−7.22, 30.39)	**9.45 (2.39, 16.51)**	9.92 (−3.49, 23.32)	8.33 (−7.44, 24.10)	8.34 (−3.42, 20.10)	8.31 (−8.03, 24.64)	4.56 (−1.38, 10.51)	2.91 (−3.56, 9.39)	TAE

The bold values represent the signify statistical significance.

### 3.5. Publication bias

As is vividly shown in Supplementary Appendix D, funnel plots were employed to detect publication bias, while no significant publication bias was revealed by the visual inspection of funnel plots for all indicators.

## 4. Discussion

In this study, motor ability, attention problems, social problems, cognitive flexibility, inhibition switching, and working memory are adopted as outcome indicators to compare the effects of different interventions on each outcome indicator. As shown in Table 8, it has been shown in our current study that perceptual-motor training, traditional aerobic exercise, as well as aquatic exercise were the top three interventions for benign development in motor ability. When it comes to attention problems, aquatic exercise, pharmacotherapy, and cognitive-motor training were the top three interventions to reduce attention problems. As for the indicator of the social problem, horsemanship, pharmacotherapy, and aquatic exercise were the top three interventions in reducing social problems. In terms of cognitive flexibility, aquatic exercise, mind-body exercise, and cognitive intervention were the top three interventions to increase cognitive flexibility. For inhibition switching, cognitive-motor training, perceptual-motor training, and combination exercise were the top three interventions to reduce inhibition switching time. Finally, in terms of working memory indicators, perceptual-motor training, pharmacotherapy, and horsemanship were the top three interventions for enhancing working memory. It has been shown in our findings that there is no single intervention most effective across all outcome indicators, and different interventions may be more effective for different outcomes.

**TABLE 8:** Ranking of SUCRA probabilities for each outcome indicator.

Intervention	Motor ability		Attention problems		Social problems	
	**Sucra**	**Rank**	**Sucra**	**Rank**	**Sucra**	**Rank**
Horsemanship	51.8	6	63.1	4	79.4	1
Combination exercise	60.9	4	40	7	/	/
Perceptual-motor training	82.7	1	/	/	/	/
Cognitive-motor training	58.1	5	68.5	3	29.3	5
Aquatic exercise	69	3	80.9	1	63.7	3
Mind-body exercise	/	/	45.1	5	/	/
Acute aerobic exercise	/	/	/	/	/	/
Traditional aerobic exercise	79.6	2	23.1	10	48.7	4
Sensory integration training	/	/	/	/	/	/
Neurofeedback	0	9	36.3	8	/	/
Cognitive intervention	/	/	28.9	9	/	/
Pharmacotherapy	15.5	8	72.4	2	64.6	2
No intervention	32.4	7	41.7	6	14.4	6
**Intervention**	**Cognitive flexibility**		**Inhibition switching**		**Working memory**	
	**Sucra**	**Rank**	**Sucra**	**Rank**	**Sucra**	**Rank**
Horsemanship	/	/	43	10	66.3	3
Combination exercise	39.1	7	71.5	3	61.2	5
Perceptual-motor training	/	/	78	2	73.3	1
Cognitive-motor training	55.7	5	83.5	1	62.3	4
Aquatic exercise	86.6	1	4.7	12	/	/
Mind-body exercise	65.7	2	48.6	7	66.9	2
Acute aerobic exercise	60	4	58	5	51.1	6
Traditional aerobic exercise	2	9	48.9	6	10.3	11
Sensory integration training	/	/	43.1	9	/	/
Neurofeedback	/	/	58.2	4	25.6	10
Cognitive intervention	60.9	3	30.1	12	51.1	7
Pharmacotherapy	53	6	47.9	8	33.4	9
No intervention	28	8	34.6	11	48.3	8

Perceptual-motor training is the best physical activity intervention for children with ADHD regarding motor ability and working memory. This type of training combines physical activities such as coordination, balance, and strength with perceptual tasks (Hattabi et al., 2019). Previous research has demonstrated a strong correlation between motor behavior and underlying perceptual processes (Chu and Reynolds, 2007). In particular, when physical activity is designed to improve attention, it will contribute to developing executive functions (Piek et al., 2004; Hung et al., 2013). By combining training activities with perceptual tasks, there is potential for an overall improvement in motor ability and working memory in children with ADHD (Mandich et al., 2001).

The aquatic exercise was the intervention with the highest frequency (4 sessions) in the top three rankings for all outcome indicators and the best physical activity intervention in terms of both attention problems and cognitive flexibility. Aquatic exercise is a form of physical activity in which the training process is completed in an aquatic environment. Due to the fluid nature of water, physical activity in an aquatic environment requires participants to constantly pay attention to the environment’s fluctuations (Vivas et al., 2011). At the same time, the buoyancy effect of water provides an auxiliary force, resistance, or support, which makes physical activity in the water environment safer, and children’s activity can be more active (Broach and Dattilo, 1996). For example, swimming in water sports is a highly coordinated and lateralized sport requiring control of the upper and lower limbs in an aquatic environment (Colgate and Lynch, 2004). This feature may allow for further activation of brain regions in the prefrontal cortex and amygdala, thus contributing to improved attentional problems and cognitive flexibility (Faw, 2003).

Horsemanship is the best physical activity intervention in terms of indicators of social problems. Horsemanship is a physical activity modality through learning activities with horses as a vehicle (Kern et al., 2011). It has been shown that because equestrian learning requires participants to establish trust and frequent interaction with the horse, it contributes to developing participants’ social competence (Hauge et al., 2014) and self-efficacy (Bizub et al., 2003). With this mutual relationship with the horse, children experience the horse’s feelings, which are then internalized in their behavior, enabling further development of empathy. This change will likely transfer to human interactions (Granados and Agis, 2011). At the same time, the horse’s rhythmic activity also improves the participants’ physiological responses to stress and impulsivity (Tyler, 1994; Jang et al., 2015).

Cognitive-motor training is the best physical activity intervention for inhibiting conversion indicators. Cognitive-motor training is an intervention that integrates cognitive and motor tasks to promote an individual’s physical and mental health (Amini et al., 2022). It has been shown that performing two or more cognitive-motor tasks simultaneously, such as computation in postural training and movement under computer games, will contribute more to improvements in cognitive domains compared to single-task training (van der Niet et al., 2016; Luder et al., 2018; Schmidt et al., 2020) while reducing reaction time (Wollesen et al., 2020). Cognitive-motor training requires participants to use both skill and cognitive effort to cope with unpredictable stimuli from the external environment (Chuang et al., 2015). Therefore, some researchers have suggested that this may improve participants’ executive functioning, including improvements in inhibitory switching (Kunstler et al., 2018; Gao et al., 2019).

In conclusion, physical activity interventions have varying levels of effect on different indicators related to the symptoms of children with ADHD. This impact is dependent on the components, characteristics, and settings of the intervention. Nevertheless, physical activity interventions have been found to have numerous advantages across multiple indicators.

## 5. Strengths and limitations

One advantage of our current study is that we are the first network meta-analysis of the effects of physical activity on symptoms related to children with ADHD, which provides some scientific reference for selecting appropriate physical activity therapy for children with ADHD. The second advantage is that this study explored the effects of different physical activities on different symptom indicators in children with ADHD, which can provide some scientific reference for targeted treatment. The third advantage is that the current study only included studies from randomized controlled trials and excluded observational and cross-sectional studies, which helped to enhance the reliability of the findings. However, our reticulated meta-analysis also has some limitations that may affect the interpretation of the results. First, the relatively small number of available studies and the limited number and sample size of studies included in the analysis makes it difficult to give a particularly robust conclusion. Second, the outcome indicators that could be included are still limited. In the future, more outcome indicators of symptoms related to children with ADHD should be included based on an adequate number of studies. Finally, findings should be interpreted with caution because of the small number of studies and the limited evidence for direct comparisons of some interventions. Relevant studies should be further expanded to provide evidence with higher confidence.

## 6. Conclusion

Our current study showed that the overall performance of aquatic exercise and perceptual-motor training was better. However, different physical activity interventions have different validity and individual differences regarding their effects on different indicators in children with ADHD. Therefore, to ensure that the most suitable physical activity intervention is chosen, it is essential to accurately assess each child’s specific ADHD symptoms before implementation.

## Author contributions

CL and DL conceived and designed the study. DL and DW collected the data. DL, DW, and WC analyzed and interpreted the data. DL drafted the manuscript. JY and WZ revised the manuscript. All authors have read and agreed to the published version of the manuscript, and contributed to the study conception and design.
