# Supplementary material for: Effects of different physical activity interventions on children with attention-deficit/hyperactivity disorder: A network meta-analysis of randomized controlled trials
Source: Front Neurosci. 2023 Mar 20;17:1139263. doi: 10.3389/fnins.2023.1139263 (PMC10067581; doi:10.3389/fnins.2023.1139263)
Supplement: Supplementary file 1 [file Data_Sheet_1.docx]

Effects of different physical activity interventions on children with Attention-deficit/hyperactivity disorder: a network meta-analysis of randomized controlled trials

**Dong Li^1^, Deng Wang^2^, Wenlai Cui^3^, Chenmu Li ^4*^**

^1,4^school of physical education, Guangzhou Sport University, Guangzhou, China

^2^LFE Research Group, Department of Health and Human Performance. Universidad Politécnica de Madrid, Martín Fierro 7, Madrid, Spain

^3^School of Dance and Martial Arts, Capital University of Physical Education and Sports, Beijing, China

*** Correspondence:**Chenmu LI
562417850@qq.com

**Appendix A**

#### Pubmed

Search: **((("Child"[Mesh]) OR ((Child[Title/Abstract]) OR (Children[Title/Abstract]))) AND (("Exercise"[Mesh]) OR ((((((((((((((((((((((((((Exercise[Title/Abstract]) OR (Exercises[Title/Abstract])) OR (Physical Activity[Title/Abstract])) OR (Activities, Physical[Title/Abstract])) OR (Activity, Physical[Title/Abstract])) OR (Physical Activities[Title/Abstract])) OR (Exercise, Physical[Title/Abstract])) OR (Exercises, Physical[Title/Abstract])) OR (Physical Exercise[Title/Abstract])) OR (Physical Exercises[Title/Abstract])) OR (Acute Exercise[Title/Abstract])) OR (Acute Exercises[Title/Abstract])) OR (Exercise, Acute[Title/Abstract])) OR (Exercises, Acute[Title/Abstract])) OR (Exercise, Isometric[Title/Abstract])) OR (Exercises, Isometric[Title/Abstract])) OR (Isometric Exercises[Title/Abstract])) OR (Isometric Exercise[Title/Abstract])) OR (Exercise, Aerobic[Title/Abstract])) OR (Aerobic Exercise[Title/Abstract])) OR (Aerobic Exercises[Title/Abstract])) OR (Exercises, Aerobic[Title/Abstract])) OR (Exercise Training[Title/Abstract])) OR (Exercise Trainings[Title/Abstract])) OR (Training, Exercise[Title/Abstract])) OR (Trainings, Exercise[Title/Abstract])))) AND ((Attention Deficit Disorder with Hyperactivity[MeSH Terms]) OR (((((((((((((((((((((((Attention Deficit Disorder with Hyperactivity[Title/Abstract]) OR (Attention Deficit Disorders with Hyperactivity[Title/Abstract])) OR (ADHD[Title/Abstract])) OR (Attention Deficit Hyperactivity Disorder[Title/Abstract])) OR (Hyperkinetic Syndrome[Title/Abstract])) OR (Syndromes, Hyperkinetic[Title/Abstract])) OR (Attention Deficit-Hyperactivity Disorder[Title/Abstract])) OR (Attention Deficit-Hyperactivity Disorders[Title/Abstract])) OR (Deficit-Hyperactivity Disorder, Attention[Title/Abstract])) OR (Deficit-Hyperactivity Disorders, Attention[Title/Abstract])) OR (Disorder, Attention Deficit-Hyperactivity[Title/Abstract])) OR (Disorders, Attention Deficit-Hyperactivity[Title/Abstract])) OR (ADDH[Title/Abstract])) OR (Attention Deficit Hyperactivity Disorders[Title/Abstract])) OR (Attention Deficit Disorder[Title/Abstract])) OR (Attention Deficit Disorders[Title/Abstract])) OR (Deficit Disorder, Attention[Title/Abstract])) OR (Deficit Disorders, Attention[Title/Abstract])) OR (Disorder, Attention Deficit[Title/Abstract])) OR (Disorders, Attention Deficit[Title/Abstract])) OR (Brain Dysfunction, Minimal[Title/Abstract])) OR (Dysfunction, Minimal Brain[Title/Abstract])) OR (Minimal Brain Dysfunction[Title/Abstract])))**

**Appendix B**


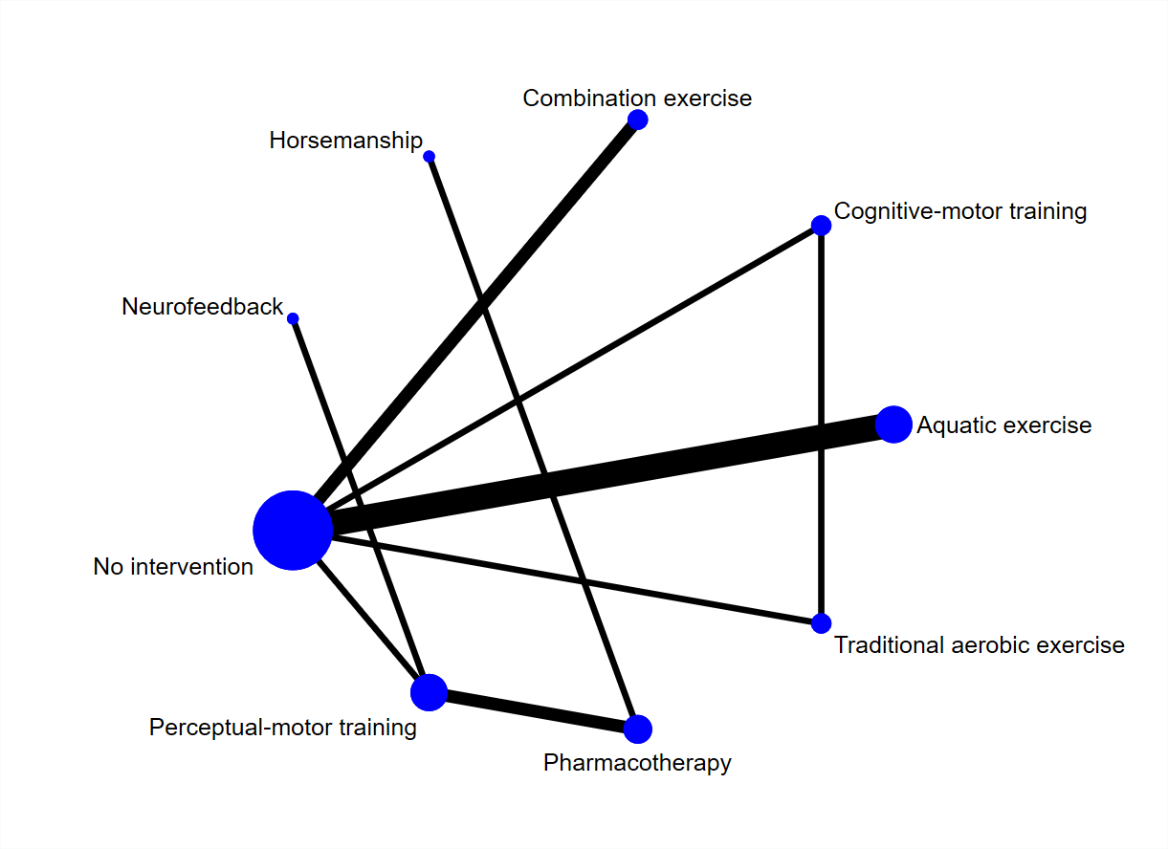


**Figure B1** Network diagram of motor ability


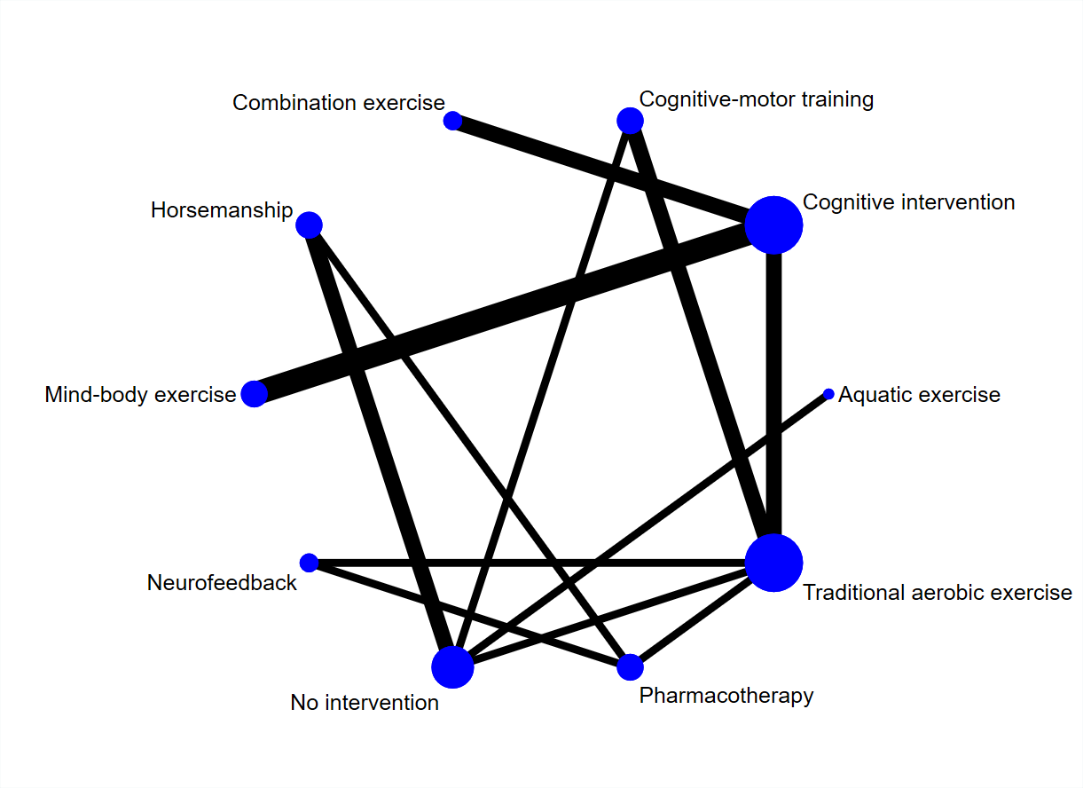


**Figure B2** Network diagram of attention problems


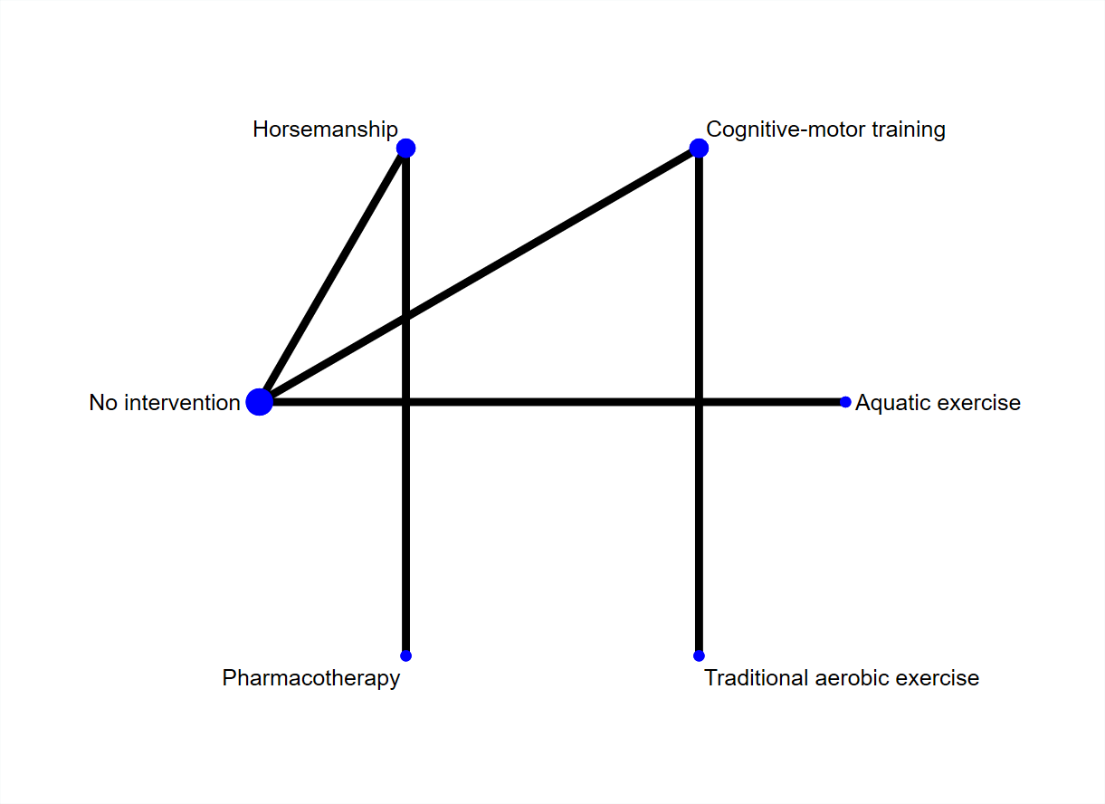


**Figure B3** Network diagram of social problems

**
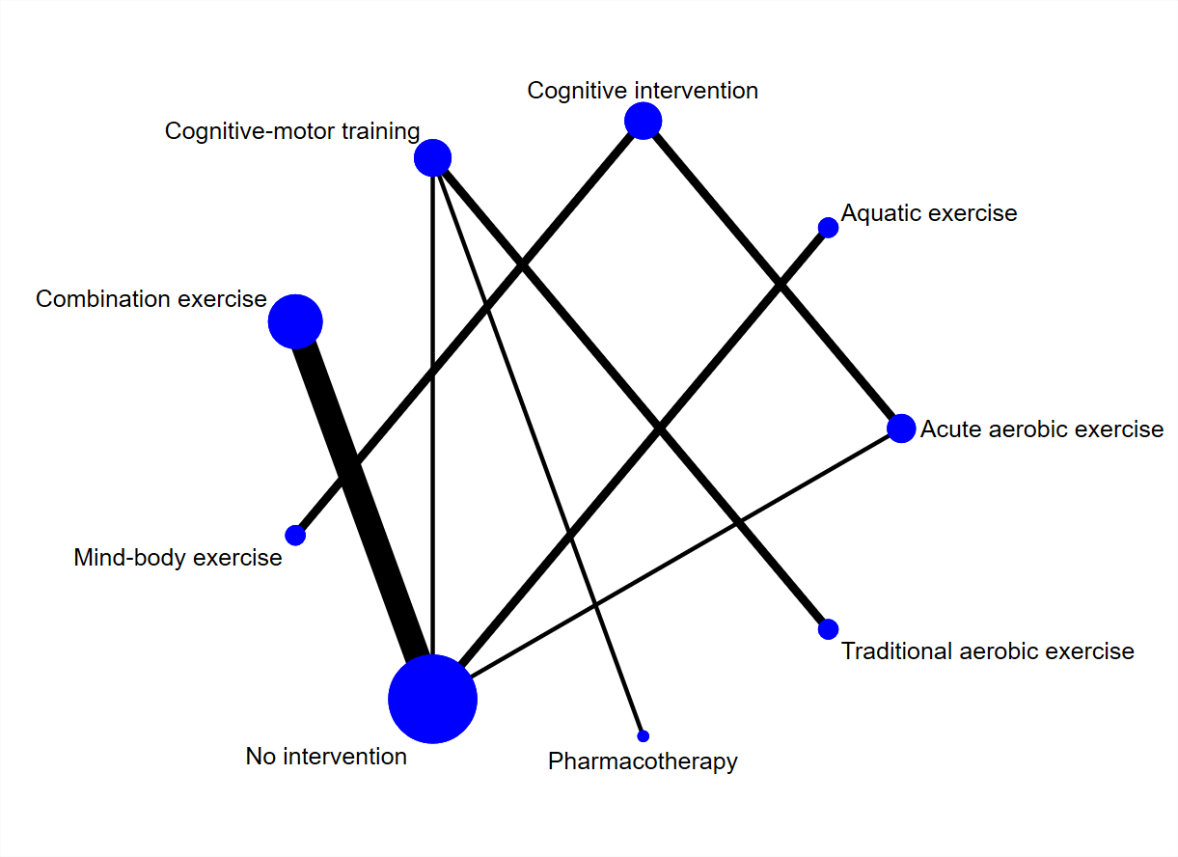
**

**Figure B4** Network diagram of cognitive flexibility


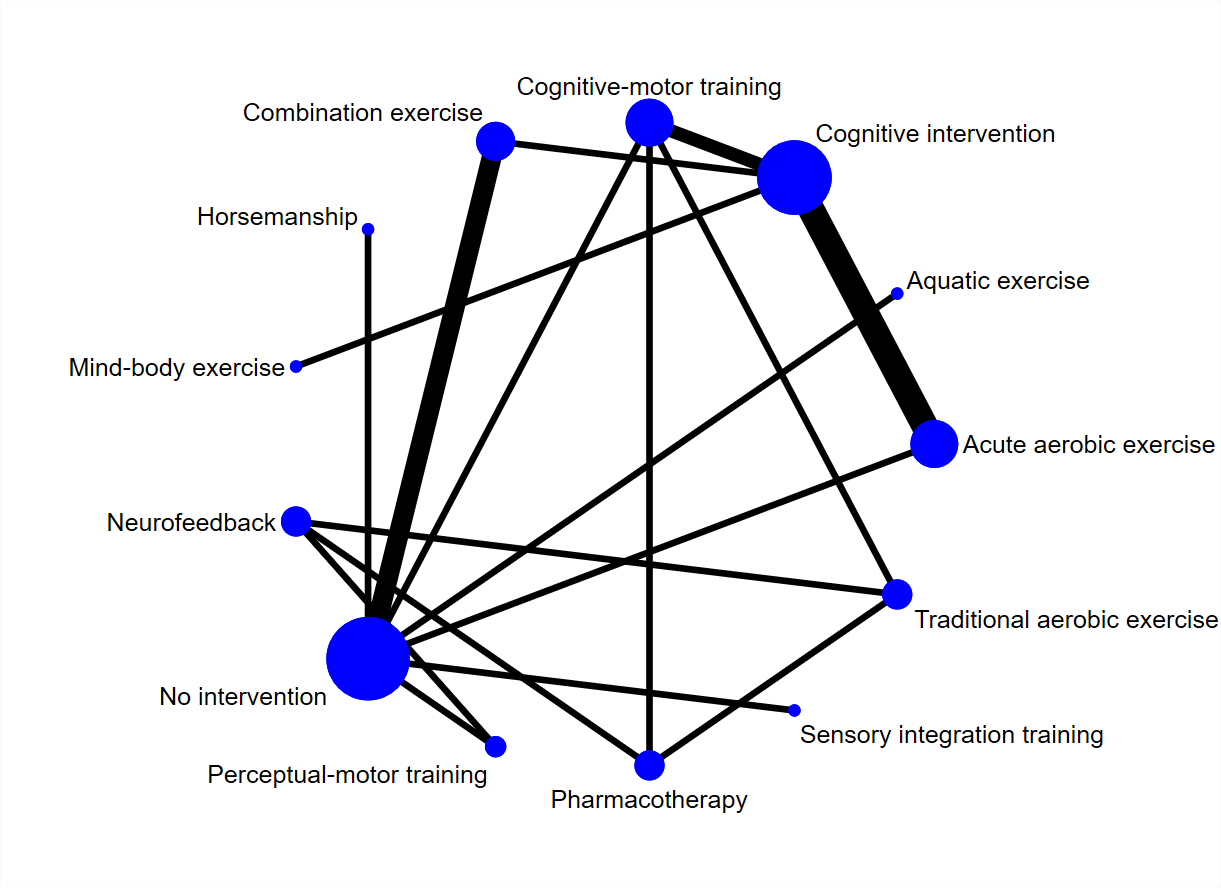


**Figure B5** Network diagram of inhibition switching


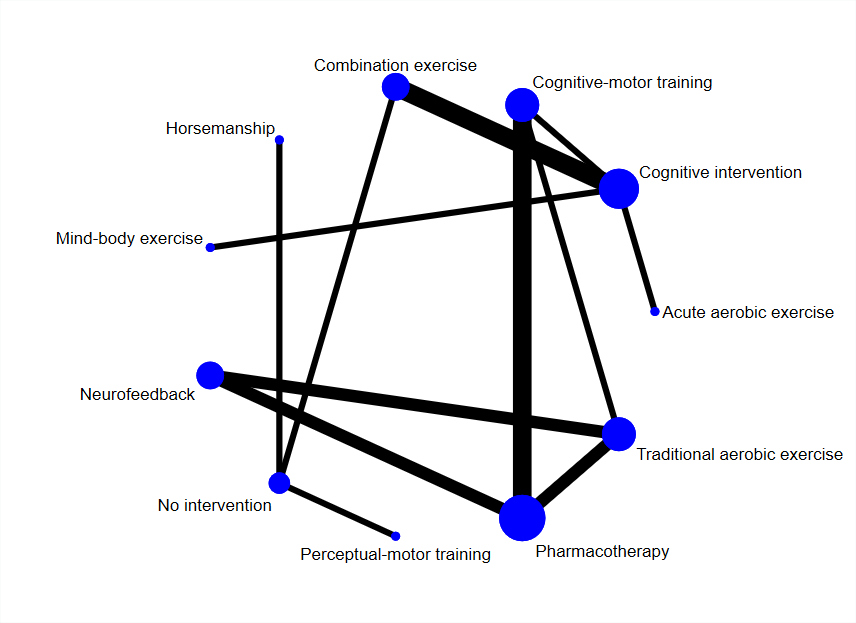


**Figure B6** Network diagram of working memory

**Appendix C**


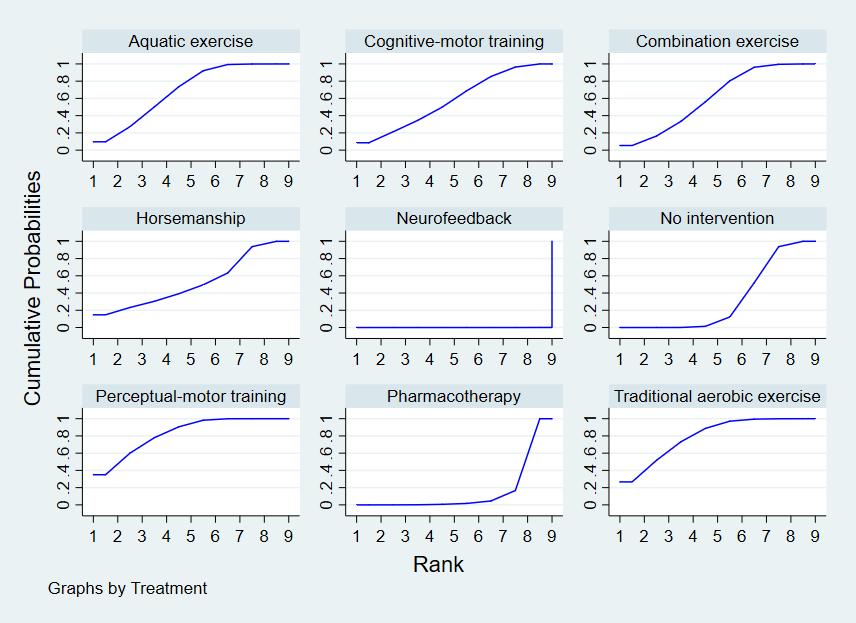


**Figure C1** SUCRA plot of motor ability


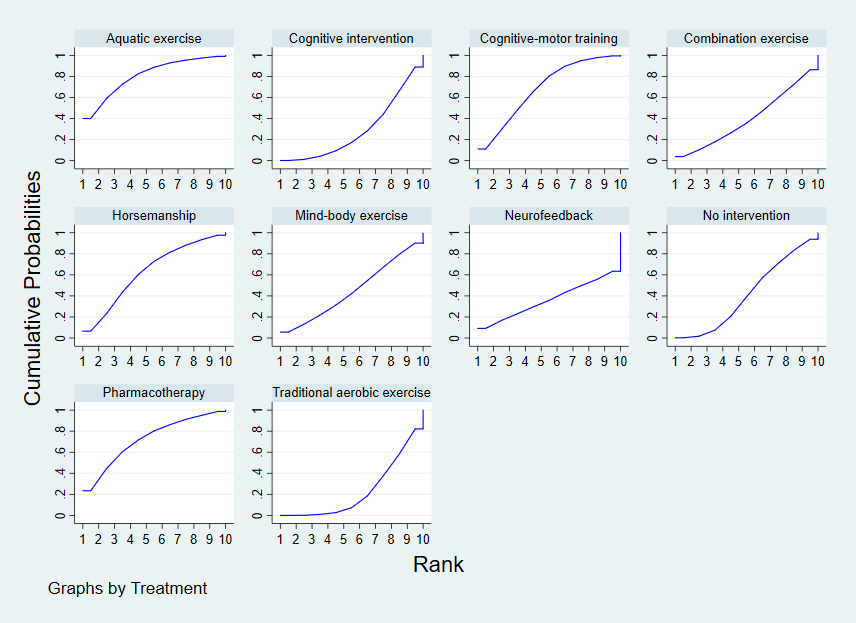


**Figure C2** SUCRA plot of attention problems


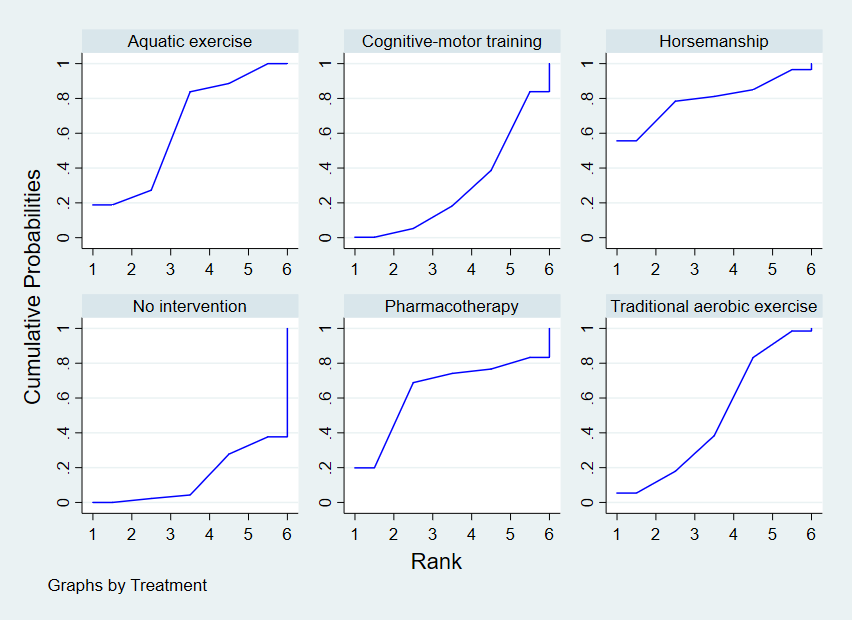


**Figure C3** SUCRA plot of social problems


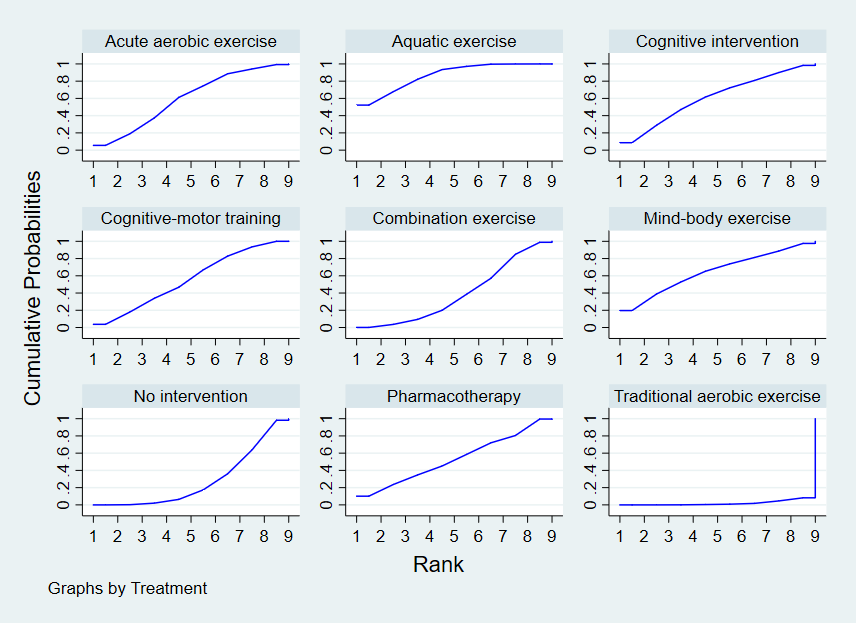


**Figure C4** SUCRA plot of cognitive flexibility


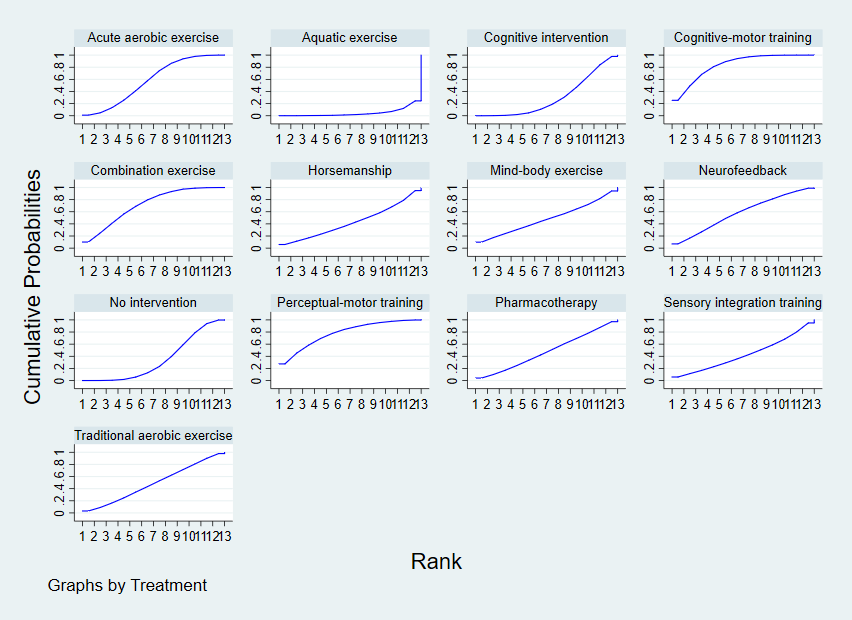


**Figure C5** SUCRA plot of inhibition switching


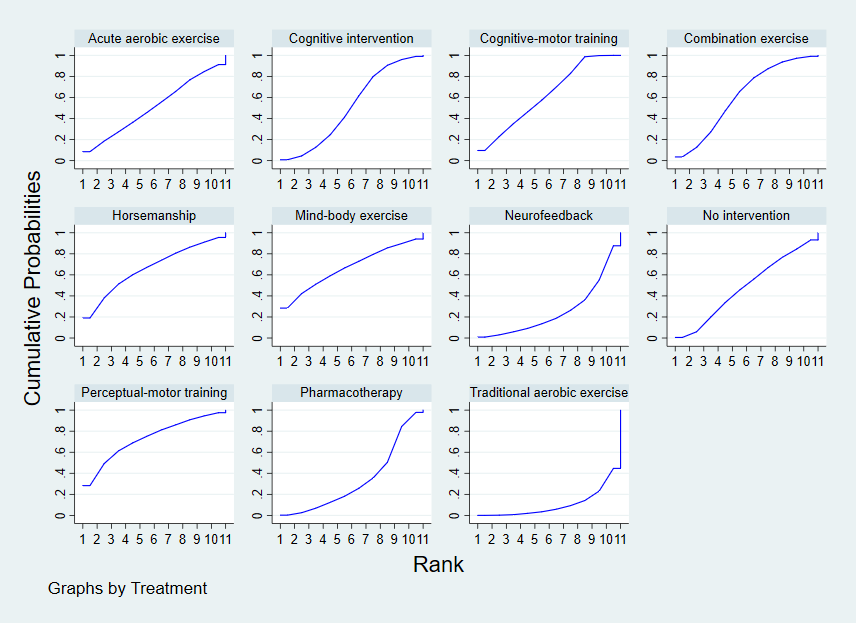


**Figure C6** SUCRA plot of working memory

**Appendix D**


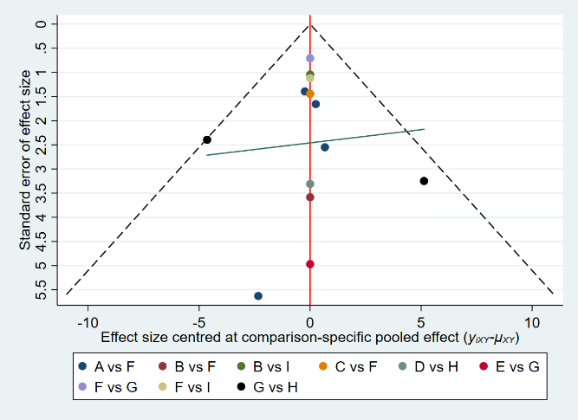

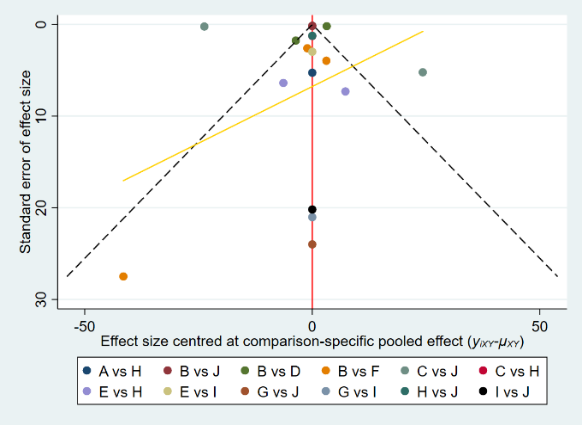


**D1**  **D2**


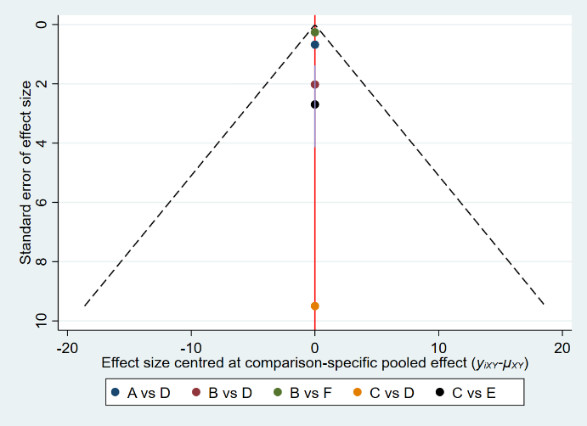

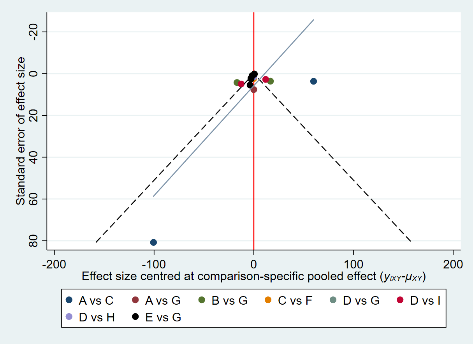


**D3**   **D4**


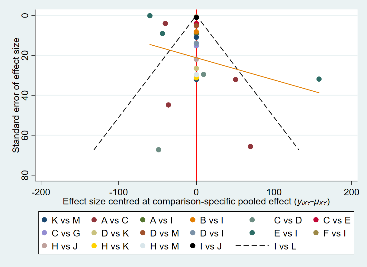

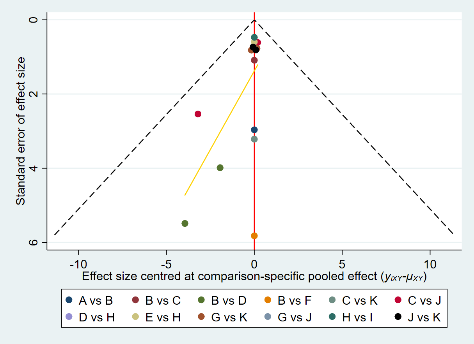


**D5**   **D6**

**Figure D.** Funnel plot on publication bias. **D1**: Motor ability; **D2**: Attention problems; **D3**: Social problems; **D4**: Cognitive flexibility; **D5**: Inhibition switching; **D6**: Working memory.
